# Rurality weakens the positive association between the COVID-19 pandemic and substance use treatment involving medications for opioid use disorder

**DOI:** 10.1016/j.dadr.2026.100408

**Published:** 2026-01-05

**Authors:** Ryan J. Lofaro, Leanne M. Confer, Robert M. Bohler, Jessica S. Schwind

**Affiliations:** a Department of Public and Nonprofit Studies at Georgia Southern University, United States; b Department of Criminal Justice and Criminology at Georgia Southern University, United States; c Jiann-Ping Hsu College of Public Health at Georgia Southern University, United States; d Institute for Health Logistics & Analytics at Georgia Southern University, United States

**Keywords:** Rurality, COVID-19, Medications for opioid use disorder (MOUD), Opioid use disorder (OUD), Treatment admissions, Treatment episode data set

## Abstract

**Introduction:**

During the COVID-19 pandemic, policies and practices were adopted to increase access to medications for opioid use disorder (MOUD), an evidence-based treatment that has lower utilization rates in rural areas compared to urban regions. However, limited attention has been given to rural-urban differences in MOUD use associated with pandemic policy and practice changes. We examine associations between the pandemic, rurality, and MOUD use in residential and outpatient treatment programs in the United States.

**Methods:**

Using 2018–2022 Treatment Episode Data Set Admissions (TEDS-A) data from residential short-term (RST), residential long-term (RLT), and outpatient treatment centers, we explored bivariate associations between MOUD as part of a treatment plan as the outcome variable and rurality and the pandemic as key independent variables. We then employed logistic regression, adjusting for multiple factors to analyze base and moderation models.

**Results:**

Findings indicated MOUD use increased across all treatment modalities in the post-COVID period with the strongest association in RLT treatment (OR = 2.298). In all treatment modalities, rurality reduced the strength of the positive relationship between the pandemic and MOUD use (interaction term ORs ranged from .441 to .91). Rural areas experienced a sharp drop in MOUD use from 2021 to 2022 in RLT and outpatient treatment.

**Conclusions:**

Gains in the use of MOUD post-pandemic appeared short-lived in rural areas, ultimately widening urban-rural disparities. Providers, policymakers, and other stakeholders should work together to sustain policies and practices that promote MOUD, particularly in rural areas.

## Highlights

• MOUD use increased across all treatment modalities in the post-COVID period.
• Rurality weakened the positive relationship between the pandemic and MOUD use.
• Rural areas experienced a sharp drop in MOUD use from 2021 to 2022.
• Providers and policymakers should work together to sustain rural MOUD access.

## 1 Introduction

Despite a recent decline in drug overdose deaths on a national scale, opioid overdose remains a leading cause of death for individuals between the ages of 18 and 44 in the United States (U.S.; CDC, 2025). During the height of the COVID-19 pandemic, there was a sharp increase in overdose deaths—driven largely by synthetic opioids—with disproportionately higher rates observed across different regions and population groups (Chen and Shen, 2022, Friedman 2023). Policies were enacted during the pandemic to reduce opioid-related harm by increasing access to medications for opioid use disorder (MOUD), such as expanding telehealth flexibilities and relaxing prescribing rules (Pessar et al., 2021). These policy changes likely increased MOUD use, although increases were modest and time-limited (Ge et al., 2024; Ali et al., 2023), and there was large variation in the implementation of these policies (Pessar et al., 2021). Of additional concern, less than a quarter of those affected by opioid use disorder (OUD) report receiving medication treatment, and among those who do receive treatment, the majority live in large metropolitan areas (Dowell et al., 2024, Jones et al., 2023; National Institute on Drug Abuse, 2023).

Rural communities have been disproportionately affected by the opioid crisis, but the impact has varied across the U.S. (Rigg et al., 2018). Despite the effectiveness of MOUD in reducing opioid-related mortality, individuals living in rural communities were significantly less likely to receive these treatments compared to their urban counterparts in the pre-pandemic period (Amiri et al., 2021, Kiang et al., 2021). Research on conditions prior to the COVID-19 pandemic indicated disparities in MOUD use across the urban-rural divide were likely due to myriad factors, including a lack of clinics/providers, physicians choosing not to prescribe, stigma, resources, and travel and cost constraints (Bresett et al., 2023; Lister et al., 2020). These pre-pandemic barriers, commonly seen in rural communities, contribute to delays in MOUD initiation rates, lower retention or completion rates, and poorer health outcomes among residents (Stopka et al., 2024). Findings from a 2021 survey of hospitals indicated that rural hospitals did not have lower odds of screening for OUD but had significantly lower odds of offering MOUD (Franz et al., 2024), though it is not known whether these conditions were exacerbated by the pandemic. The literature reveals the multifaceted nature of MOUD use across the urban-rural divide and highlights the need for comprehensive analyses to identify broad patterns, especially in the context of crisis-related disruptions.

The COVID-19 pandemic caused many health-related disparities to not only come to light but also widen (Shah et al., 2020). Recognizing that OUD was rapidly increasing during the social distancing period of the pandemic, policies and procedures were quickly implemented to maintain access and reduce barriers to essential services, such as MOUD, particularly in rural areas where distance and workforce shortages have long posed significant challenges (Jones et al., 2021, Uscher-Pines et al., 2020; SAMHSA, 2020). Thus, the COVID-19 pandemic provided a unique opportunity to examine how period-specific changes impacted MOUD use specifically in medically underserved rural populations. Several studies found COVID-19 telehealth policies influenced MOUD treatment in rural areas by increasing virtual visit use, affecting retention patterns based on treatment initiation timing, and revealing both high acceptability and implementation challenges due to infrastructure and workforce limitations (Calhoun et al., 2024, Hughes et al., 2021, Ober et al., 2024). Several studies have identified the complexities that exist between policies, healthcare infrastructure, and rural community culture (Jalali et al., 2020, Lister et al., 2020, Thomas et al., 2020). However, significant gaps remain in understanding how rurality and pandemic-era disruptions intersect to influence MOUD uptake on a nationwide scale. Specifically, there is limited research on whether the pandemic widened or narrowed urban-rural disparities in MOUD uptake and whether rural changes were sustained in the post-pandemic period.

In addition to rurality and the pandemic, treatment modality plays a critical role in access to MOUD. While residential treatment provides a structured environment and housing for individuals receiving services (Reif et al., 2014), residential patients are consistently less likely to use MOUD compared to their outpatient counterparts (Beetham et al., 2020, Hartung et al., 2022, Lofaro et al., 2025, Solomon et al., 2022). Potential reasons include the tendency of residential treatment programs to embrace a 12-step abstinence-based treatment philosophy that may be viewed as inconsistent with recovery pathways involving MOUD (Huhn et al., 2020). In addition, facilities offering short-term residential treatment are more likely to accept patients using MOUD and to prescribe buprenorphine to treat OUD, while long-term residential treatment facilities show no such pattern (Nichols et al., 2024), suggesting differences across residential treatment modalities. Pandemic-related disparities across treatment modalities are also apparent. Following the onset of COVID-19, residential treatment programs struggled due to fewer clients, staff layoffs and fatigue, delayed treatment initiation, reduced treatment services, and difficulties with telehealth (Pagano et al., 2021), whereas disruptions to outpatient OUD and MOUD treatment were less prominent, as providers pivoted to telehealth services in environments not requiring residential living or face-to-face interactions (Tormohlen et al., 2024). Such disruptions may be exacerbated in rural areas, given the provider shortages, reduced prescribing rates, increased stigma, and lack of resources (Bresett et al., 2023; Lister et al., 2020).

Grounded in the understanding that MOUD-related practices have changed following the pandemic (Kline et al., 2023, Pessar et al., 2021), including in rural areas (Calhoun et al., 2024, Ober et al., 2024), as well as findings on urban-rural disparities in MOUD use more generally (Andrilla et al., 2018; Bresett et al., 2023; Lister et al., 2020), we asked the following research questions: Does the use of MOUD as part of a treatment plan change across pre- and post-COVID periods in residential and outpatient treatment programs? Does rurality moderate the relationship between the pandemic and MOUD uptake? This study adds to the literature by providing a nationwide analysis of changes to MOUD use in various treatment settings across the U.S. with a focus on pandemic- and rurality-associated variations. We contribute to previous studies by highlighting how treatment practices that increased the use of MOUD in the post-pandemic period largely benefited urban areas and thus widened extant urban-rural disparities, as pandemic-associated increases in MOUD utilization in rural areas were short-lived.

## 2 Method

### 2.1 Dataset

We leveraged the Substance Abuse and Mental Health Services Administration’s (SAMHSA’s) Treatment Episode Dataset Admissions (TEDS-A) database to capture admissions to publicly funded substance use treatment programs in the U.S. To analyze those most likely to be prescribed MOUD as part of a treatment plan, we included treatment admissions whose primary substance was either heroin, non-prescription methadone, or other opiates and synthetics. The sample included individuals aged 18 and over who entered treatment in 2018, 2019, 2021, or 2022. We excluded 2020 because *year* was the most granular level of temporality in the TEDS-A data, so discerning between pre- and post-pandemic 2020 was not possible. To compare findings across treatment types, our sample included patients who entered residential short-term (RST), residential long-term (RLT), or outpatient treatment, given variations in the use of MOUD across these modalities (Hartung et al., 2022). Aligned with past studies, outpatient treatment includes ambulatory non-intensive and intensive state-licensed substance use treatment centers to gain a broad understanding of settings where MOUD uptake is most likely to occur (Butelman et al., 2025, Hartung et al., 2022, Lofaro et al., 2025) and compare this to various residential modalities. Consistent with prior research utilizing TEDS data (Mennis et al., 2019, Sahker et al., 2015, Turvey et al., 2020), we included only those with no prior treatment admissions to reduce biases associated with duplicate treatment admissions (Bormann and Arndt, 2024). We excluded Puerto Rico because it accounted for less than 1 % of the dataset.

### 2.2 Measures

#### 2.2.1 Dependent variable: treatment involving medications for opioid use disorder

MOUD was a binary variable that indicated whether the use of medications, such as methadone, buprenorphine, and/or naltrexone, was a part of the patient’s treatment plan at admission. Though TEDS does not provide data on the type of MOUD, past studies included this variable in analyses to gain a general understanding of MOUD use in treatment centers (Askari et al., 2020, Butelman et al., 2025, Krawczyk et al., 2021, Stahler et al., 2021).

#### 2.2.2 Independent variables: rurality and COVID-19

We operationalized rurality based on the Core Based Statistical Area (CBSA) codes contained in the TEDS data. Metropolitan or micropolitan areas with an urban core population of at least 10,000 people based on Census data were considered urban while those without a CBSA code were considered rural. Past studies used the same methods to capture urban-rural disparities (Gilbert et al., 2024, Lofaro et al., 2025, Stahler et al., 2021, Turvey et al., 2020).

For the COVID-19 variable, we included 2018, 2019, 2021, and 2022 to analyze a balanced number of years pre- and post-pandemic with the most recent data from TEDS at the time of the analysis. The main analysis included an independent variable coded as 1 =  post-COVID-19 for treatment admissions in 2021 and 2022 and as 0 =  pre-COVID-19 for treatment admissions in 2018 and 2019.

#### 2.2.3 Control variables

Analyses incorporated several covariates included as controls in past studies on MOUD (Donahoe et al., 2024; Presskreischer et al., 2025; Welsh et al., 2023). Our multivariable analyses accounted for the patient’s race/ethnicity, age, gender, education, employment status, living arrangement, referral source, and arrests in the preceding 30 days. We also controlled for region to account for the spatial effects of rurality (see Lofaro et al., 2025; Rigg et al., 2018). These geographic divisions were based on Census definitions and split into the Northeast, Midwest, South, and West. Models did not include insurance status due to this variable missing a substantial amount of data (51.97 % missing). As a robustness check, we included this variable as a control in analyses in the appendix.

### 2.3 Analysis

Findings were derived from both bivariate and multivariable analyses. We first conducted Chi-square tests of independence to analyze associations between COVID-19 time periods and MOUD, as well as urban-rural differences between the time periods. Then, we ran logistic regression models, given the dichotomous nature of MOUD, to analyze associations between COVID-19, rurality, and MOUD with base models and those with an interaction term between COVID-19 and rurality. Additional analyses examined similar moderation models but replaced the COVID-19 variable with year of admission and used margins plots to better understand temporal patterns at a more granular level. All analyses were disaggregated by treatment modality (RST, RLT, and outpatient).

## 3 Results

### 3.1 Descriptive statistics

Table 1 contains descriptive statistics for the full sample and each treatment type. Approximately half of treatment admissions used MOUD as part of their treatment plan (49.83 %), though this varied greatly across modalities, as 15.18 % of RST admissions used MOUD compared to 9.8 % in RLT and 57.32 % in outpatient treatment. The majority of admissions occurred in the pre-COVID period (66.37 %), with consistent patterns across treatment types. Urban areas comprised the majority of admissions (77.95 %). Comparing across modalities, rural regions had the most representation in outpatient treatment (22.76 %) and smallest in RLT (16.44 %). In the full sample, the majority of admissions were White non-Hispanic (64.18 %), were male (60.43 %), had a high school graduate degree or GED (52.97 %), were referred by an individual (62.57 %), lived independently (69.36 %), and had zero arrests in the past 30 days (93.48 %). A plurality were aged 25–34 (40 %), unemployed (41.92 %), and entering treatment in the West (35.07 %).

**Table 1:** Descriptive statistics by treatment modality.

	Residential Short-Term (n = 29,981)	Residential Long-Term (n = 26,375)	Outpatient (n = 279,730)	Total (n = 336,086)
	%(n)	%(n)	%(n)	%(n)
MOUD				
No	84.82 % (25,430)	90.20 % (23,790)	42.68 % (119,381)	50.17 % (168,601)
Yes	15.18 % (4551)	9.80 % (2585)	57.32 % (160,349)	49.83 % (167,485)
Pandemic				
Pre-COVID	63.59 % (19,064)	66.26 % (17,477)	66.68 % (186,513)	66.37 % (223,054)
Post-COVID	36.41 % (10,917)	33.74 % (8898)	33.32 % (93,217)	33.63 % (113,032)
Rurality				
Urban	79.56 % (23,852)	83.56 % (22,039)	77.24 % (216,073)	77.95 % (261,964)
Rural	20.44 % (6129)	16.44 % (4336)	22.76 % (63,657)	22.05 % (74,122)
Race/Ethnicity				
White Non-Hispanic	68.91 % (20,660)	59.62 % (15,724)	64.10 % (179,320)	64.18 % (215,704)
Black Non-Hispanic	16.06 % (4816)	9.57 % (2523)	13.49 % (37,724)	13.41 % (45,063)
Other Race Non-Hispanic	4.35 % (1305)	8.22 % (2168)	5.34 % (14,930)	5.48 % (18,403)
Hispanic	10.67 % (3200)	22.60 % (5960)	17.07 % (47,756)	16.93 % (56,916)
Age				
18–24	14.89 % (4464)	13.97 % (3684)	8.94 % (25,012)	9.87 % (33,160)
25–34	44.42 % (13,319)	48.74 % (12,856)	38.70 % (108,250)	40.00 % (134,425)
35–44	23.56 % (7065)	22.42 % (5913)	25.48 % (71,280)	25.07 % (84,258)
45–54	10.77 % (3229)	9.48 % (2501)	14.52 % (40,627)	13.79 % (46,357)
55 +	6.35 % (1904)	5.39 % (1421)	12.36 % (34,561)	11.27 % (37,886)
Gender				
Male	63.72 % (19,105)	63.39 % (16,720)	59.80 % (167,273)	60.43 % (203,098)
Female	36.28 % (10,876)	36.61 % (9655)	40.20 % (112,457)	39.57 % (132,988)
Education				
<12	24.38 % (7309)	25.96 % (6847)	24.29 % (67,949)	24.43 % (82,105)
12 or GED	52.16 % (15,637)	49.62 % (13,086)	53.38 % (149,310)	52.97 % (178,033)
> 12	23.46 % (7035)	24.42 % (6442)	22.33 % (62,471)	22.60 % (75,948)
Employment Status				
Full Time	10.86 % (3255)	4.29 % (1132)	19.04 % (53,252)	17.15 % (57,639)
Part Time	2.82 % (845)	1.94 % (511)	8.39 % (23,474)	7.39 % (24,830)
Unemployed	50.64 % (15,181)	40.85 % (10,773)	41.09 % (114,928)	41.92 % (140,882)
Not in Labor Force	35.69 % (10,700)	52.93 % (13,959)	31.49 % (88,076)	33.54 % (112,735)
Referral Source				
Individual	48.71 % (14,605)	40.92 % (10,793)	66.10 % (184,891)	62.57 % (210,289)
Alcohol/Drug Use Care Provider	14.15 % (4241)	12.55 % (3309)	4.49 % (12,568)	5.99 % (20,118)
Other Healthcare Provider	7.93 % (2378)	5.28 % (1393)	5.18 % (14,494)	5.43 % (18,265)
School	0.07 % (21)	0.01 % (3)	0.07 % (197)	0.07 % (221)
Employer	0.30 % (91)	0.08 % (22)	0.16 % (451)	0.17 % (564)
Other Community Referral	8.53 % (2557)	15.14 % (3993)	8.43 % (23,589)	8.97 % (30,139)
Court/CJ/DWI/DUI	20.31 % (6088)	26.02 % (6862)	15.57 % (43,540)	16.81 % (56,490)
Living Arrangements				
Homeless	20.49 % (6144)	38.87 % (10,251)	9.33 % (26,107)	12.65 % (42,502)
Dependent Living	20.25 % (6071)	26.37 % (6954)	16.96 % (47,433)	17.99 % (60,458)
Independent Living	59.26 % (17,766)	34.77 % (9170)	73.71 % (206,190)	69.36 % (233,126)
Past Arrests				
0	89.25 % (26,757)	88.31 % (23,292)	94.43 % (264,140)	93.48 % (314,189)
1	9.32 % (2795)	10.16 % (2680)	4.42 % (12,364)	5.31 % (17,839)
2 +	1.43 % (429)	1.53 % (403)	1.15 % (3226)	1.21 % (4058)
Region				
Northeast	28.86 % (8653)	14.24 % (3755)	20.84 % (58,308)	21.04 % (70,716)
Midwest	21.11 % (6328)	11.00 % (2901)	13.60 % (38,033)	14.06 % (47,262)
South	43.67 % (13,093)	16.41 % (4328)	29.60 % (82,814)	29.82 % (100,235)
West	6.36 % (1907)	58.35 % (15,391)	35.95 % (100,575)	35.07 % (117,873)

### 3.2 Bivariate associations

**Fig. 1:**
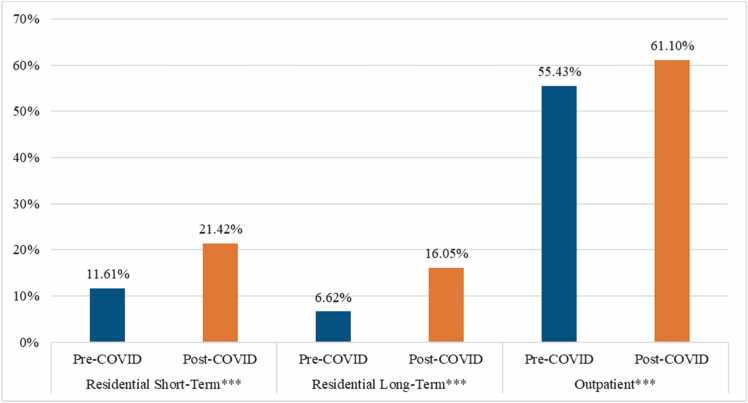
Percentage of Treatment Admissions Using MOUD Across Pre- and Post-COVID Periods by Treatment Modality. ^⁎^*p* < 0.05, ^⁎⁎^*p* < 0.01^, ⁎⁎⁎^*p* < 0.001.

Fig. 1 shows results for bivariate associations between COVID and MOUD by treatment modality. Across all treatment types, Chi-square tests of independence were significant (*p* < .001), showing consistent increases in MOUD use in the post-COVID period. The largest differences across periods were in RLT and RST treatment, wherein, respectively, MOUD rates more than doubled (16.05 % against 6.62 %) and almost doubled (21.42 % against 11.61 %) from pre- to post-COVID-19 time periods. In outpatient treatment, MOUD rates increased by 5.67 percentage points from 55.43 % to 61.1 %.

**Fig. 2:**
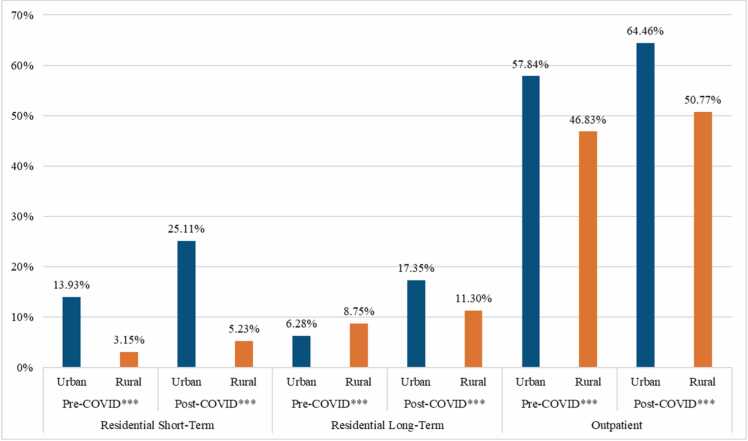
Percentage of Treatment Admissions Using MOUD Across Urban and Rural Catchments by Treatment Modality and Pre- and Post-COVID Periods. ^⁎^*p* < 0.05, ^⁎⁎^*p* < 0.01^, ⁎⁎⁎^*p* < 0.001.

Fig. 2 displays results similar to those in Fig. 1 but examines urban-rural disparities from pre- to post-COVID. Across all treatment modalities and time periods, there were significant differences between urban and rural areas (*p* < .001). Results show the pandemic widened the urban-rural MOUD gap. The largest disparity emerged in RST treatment; in the pre-COVID period, urban MOUD rates outpaced rural rates by 10.78 percentage points, and this grew to 19.88 percentage points in the post-COVID period. The smallest change appeared in outpatient treatment (11.01 percentage point difference pre-pandemic and 13.69 percentage point difference post-pandemic). In RLT treatment prior to COVID, rural facilities had increased MOUD use compared to urban treatment centers, with a delta of 2.47 percentage points, but following COVID, urban facilities outperformed their rural counterparts by 6.05 percentage points.

### 3.3 Regression models

Base and moderation models for associations between rurality, COVID-19, and MOUD are presented in Table 2, with analyses disaggregated by treatment modality and adjusted for covariates. Base models confirmed results shown in Fig. 1, Fig. 2. When holding other factors fixed, MOUD rates increased in the post-COVID-19 period relative to pre-COVID-19 and decreased in rural regions relative to urban areas. Rurality had the largest association in RST treatment, wherein treatment admissions in rural regions had 76 % lower odds of using MOUD as part of their treatment plan. Regarding the COVID-19 variable, the largest association occurred for RLT treatment patients; the odds of MOUD use were 2.3 times higher in the post-COVID-19 period.^1^

**Table 2:** Base and moderation logit regression models analyzing associations between COVID, rurality, and MOUD use, split by treatment modality.

	Residential Short-Term		Residential Long-Term		Outpatient	
	Base	Moderation	Base	Moderation	Base	Moderation
*Rural*	0.266^⁎⁎⁎^	0.406^⁎⁎⁎^	0.878^⁎^	1.159	0.844^⁎⁎⁎^	0.872^⁎⁎⁎^
	(0.231, 0.307)	(0.336, 0.490)	(0.772, 0.999)	(0.971, 1.383)	(0.826,0.863)	(0.849, 0.896)
*COVID*	2.064^⁎⁎⁎^	2.180^⁎⁎⁎^	2.298^⁎⁎⁎^	2.525^⁎⁎⁎^	1.211^⁎⁎⁎^	1.241^⁎⁎⁎^
	(1.922, 2.215)	(2.026, 2.346)	(2.079, 2.540)	(2.264, 2.816)	(1.188,1.235)	(1.214, 1.269)
*Rural x COVID*		0.441^⁎⁎⁎^		0.578^⁎⁎⁎^		0.910^⁎⁎⁎^
		(0.333, 0.584)		(0.450, 0.742)		(0.871, 0.951)
*N*	29,981	29,981	26,375	26,375	279,730	279,730

Note: Odds Ratios Displayed; 95 % confidence intervals in parentheses; Models adjusted for race and ethnicity, age, gender, education, employment, living arrangement, referral source, recent arrests, and region

^⁎^*p* < 0.05, ^⁎⁎^*p* < 0.01, ^⁎⁎⁎^*p* < 0.001

The moderation models showed that rural areas were unable to capitalize on the improvements in MOUD use seen in urban regions. In RST and outpatient treatment programs, rural regions had less MOUD use pre-COVID-19, and MOUD use increased in urban areas post-COVID-19, but rural areas did not observe this same increase, meaning disparities grew larger between the catchment types. In RLT treatment, though there was no significant rural disadvantage pre-COVID-19, the pandemic was associated with disparities, as rural areas did not see the same improvements in MOUD use as urban areas did. The rural disadvantage was most severe in RST treatment (OR =.441, *p* < .001) and least severe in outpatient treatment (OR =.91, *p* < .001).

### 3.4 Results by year

**Fig. 3:**
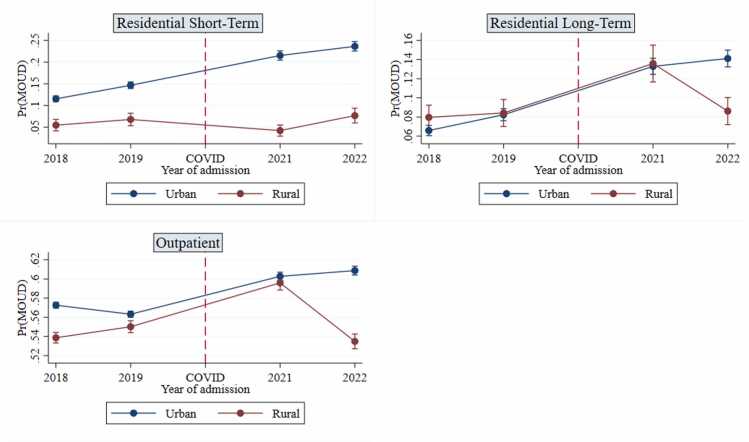
Predicted Margins of MOUD Use by Year and Rurality, Split by Treatment Modality.

To explore which of the individual years may be driving results, we ran moderation models including *year of admission* (coded as 2018, 2019, 2021, 2022) in lieu of the COVID-19 variable. Fig. 3 shows post-estimation margins plots for these regression models (see Table A2 of appendix for regression table). In urban areas, there was a general increase across years in all treatment types. Regarding rural regions, there was an increase in MOUD use from 2019 to 2021 in RLT and outpatient treatment. However, these modalities experienced a sharp decrease from 2021 to 2022. More specifically, the predicted probability of receiving MOUD in RLT treatment in rural regions dropped from 13.6 % to 8.6 % in the post-COVID-19 years, and in outpatient treatment, the drop was from 59.6 % to 53.5 %. MOUD use in rural RST treatment stayed consistent throughout the four years.^2^

## 4 Discussion

This study examined how the COVID-19 pandemic and attending treatment in rural areas affected MOUD utilization in different treatment settings across the U.S. from 2018 to 2022. After the pandemic began, MOUD use increased in all types of treatment, especially in short- and long-term residential treatment programs. However, rurality reduced the strength of the positive effects of COVID on MOUD usage. The pandemic thus widened urban-rural disparities in the use of this recovery pathway. When examining the post-pandemic period by year, any increases in MOUD utilization in rural areas were short-lived, dropping back to pre-pandemic levels in RLT and outpatient treatment settings.

Our findings on MOUD use during the pandemic are aligned with previous studies on the expansion of MOUD access during this period, in which policies were enacted in the U.S. to allow for buprenorphine prescriptions via telehealth appointments, relax buprenorphine prescribing requirements, and increase flexibility in take-home methadone (Pessar et al., 2021). Studies have shown, relative to in-person initiation, telehealth initiation of MOUD increased in 2020, 2021, and 2022 (Ge et al., 2024), and there were increases in the number of patients prescribed buprenorphine in the early phases of the pandemic (Ali et al., 2023). Our results extend these findings to demonstrate that increases in MOUD use were also present in RST, RLT, and outpatient treatment. However, pandemic-related effects were not uniform across all modalities, as the largest growth was observed in residential treatment facilities, possibly because this treatment modality had lower baseline MOUD levels, consistent with previous studies (Hartung et al., 2022, Solomon et al., 2022). Such an increase provides positive evidence for treatment providers that aim to increase the low proportion of patients with OUD who use MOUD (Dowell et al., 2024, Jones et al., 2023) and reverse the rise in opioid overdose deaths that occurred following the pandemic (Chen and Shen, 2022, Friedman 2023). As observed in urban areas, pandemic-related changes can be sustained and built upon to ensure people who use opioids are given access to evidence-based treatment.

Rural regions, however, did not experience the same positive pandemic-related effects as their urban counterparts and were less likely to utilize MOUD overall. Rural treatment centers were less likely to include MOUD as part of a treatment plan, consistent with past research documenting barriers to this treatment pathway, such as stigma, limited provider availability, and transportation challenges (Amiri et al., 2021; Bresett et al., 2023; Kiang et al., 2021; Lister et al., 2020). Insufficient access to this evidence-based treatment in rural areas may hinder individuals with OUD from receiving the care necessary to reduce overdose risk and support sustained recovery. The magnitude of this disparity in our study underscores the severity of the issue, especially in RST treatment settings where rural admissions had one-quarter the odds of receiving MOUD compared to their urban counterparts. This indicates that rural areas are experiencing particularly poor MOUD utilization within treatment modalities that already demonstrate lower adoption rates (Hartung et al., 2022, Solomon et al., 2022).

One of the central contributions of the current study includes examining the intersection of rurality and the pandemic to find that rural regions did not capitalize on pandemic-related gains in the use of MOUD as part of a treatment plan in RST, RLT, and outpatient treatment. Rural treatment centers not only had reduced MOUD use prior to the pandemic but also experienced smaller increases in the post-pandemic period relative to their urban counterparts. In other words, our findings illustrate that rurality dampened the positive effects of the pandemic on MOUD rates, thus widening urban-rural disparities. Such findings align with past studies that have highlighted the MOUD-related challenges rural regions faced in the post-pandemic period as the result of limited internet connectivity, communication gaps, and workforce shortages, among other factors (Calhoun et al., 2024, Ober et al., 2024). By examining the moderating effect of rurality, our study provides the first demonstration of how pandemic-related practices in the U.S. expanded extant urban-rural disparities rather than ameliorating them.

Even in the RLT and outpatient rural treatment settings where MOUD use increased in 2021 relative to 2018 and 2019, these benefits were short-lived, as sharp decreases occurred from 2021 to 2022, which returned MOUD rates in rural regions to pre-pandemic levels. This suggests that pandemic-related policies and practices to increase MOUD did not lead to sustained utilization increases in rural areas. These findings corroborate work that has highlighted the temporary nature of MOUD-related benefits associated with the pandemic (Ali et al., 2022; Socias et al., 2023) while expanding upon this line of research by illustrating how rural catchments were most affected by such fleeting benefits in RLT and outpatient public treatment programs across the entire U.S. These temporal patterns reveal novel findings about short-lived policy benefits in rural areas, and future research should explore why policy changes aimed at increasing MOUD utilization may not have led to sustainable improvements.

Regarding practical implications, this study highlights the need to focus on improving MOUD access in rural treatment centers to increase evidence-based practices, especially in RST and RLT treatment centers where urban-rural disparities were the largest. Strategies may include increasing funding for MOUD-related programs, tying insurance reimbursement to MOUD accessibility, educating treatment providers on MOUD to reduce stigma, enhancing the treatment workforce in rural catchments, and improving treatment infrastructure (Andrilla et al., 2019, Hernandez-Vallant and Hurlocker, 2024, Stopka et al., 2024, Winstanley and McPherson, 2024). It is essential rural regions sustain the progress made in MOUD utilization resulting from crisis-driven policies and practices. Additionally, the study findings underscore the potential for public health emergencies to enhance access to evidence-based treatment. The fact rural areas experienced growth—albeit short-lived—demonstrates that MOUD-related enhancements can occur, but this progress must be sustained with concerted effort and strategies aimed at continuing to provide access to this evidence-based practice. Addressing barriers to MOUD use and associated social determinants of health can help improve the sustainability of rural MOUD care.

### 4.1 Limitations

This research has limitations that should be considered when interpreting the findings. Most notably, TEDS captures data only from publicly funded substance use treatment facilities and therefore does not represent the complete picture of MOUD utilization across the U.S., particularly in settings like private doctor’s offices. TEDS also represents a cross-sectional look at admissions in general and not individuals, which prompted us to limit our dataset to those without prior admissions to reduce the impact of multiple admissions of the same individuals. A longitudinal analysis exploring the impact of multiple admissions of the same individual and how rurality intersects with treatment selection should be conducted in future studies to better understand the complexities associated with service delivery. Additionally, we were limited by TEDS’s operationalization of MOUD, which did not disaggregate by type of medication used. Future research may explore if there were differences across medication type. Further, due to the variations in the pandemic across months of 2020, we decided to not include 2020 data to limit misclassification bias. If data were available by months, a more nuanced analysis could be used to explore differences as the pandemic unfolded. Finally, our study examined MOUD initiation by treatment providers and did not take into account other important clinical outcomes, such as medication retention or adherence rates that help describe the full continuum of care. Nevertheless, the use of a large, multi-year national dataset provided valuable insights into rural disparities in MOUD access, particularly as they evolved across the COVID-19 pandemic.

## 5 Conclusion

Despite these limitations, our study makes several important contributions to the literature and has implications for practitioners and policymakers. We found that urban regions had significantly higher enrollment in MOUD treatment than their rural counterparts. While MOUD treatment increased after the peak of the COVID-19 pandemic, this shift was not fully realized in rural areas beyond a temporary increase followed by an eventual decline. In contrast, urban regions sustained long-term growth. The pandemic thus widened MOUD-related disparities between urban and rural areas. Given the impact of the opioid crisis on rural areas of the U.S, the disparity in MOUD access between urban and rural treatment centers is of particular concern, especially amid growing divergence as evidenced in the current study. Additional research is needed to further explore potential explanations for these differences, including financial considerations, stigma, and/or provider willingness to prescribe MOUD at treatment centers, and how disparities in MOUD treatment may contribute to variation in community health outcomes.

## CRediT authorship contribution statement

**Ryan J. Lofaro:** Writing – review & editing, Writing – original draft, Visualization, Supervision, Methodology, Investigation, Formal analysis, Data curation, Conceptualization. **Leanne M. Confer:** Writing – review & editing, Writing – original draft, Validation, Methodology, Investigation, Conceptualization. **Robert M. Bohler:** Writing – review & editing, Writing – original draft, Validation, Methodology, Formal analysis, Conceptualization. **Jessica S. Schwind:** Writing – review & editing, Writing – original draft, Validation, Methodology, Formal analysis, Data curation, Conceptualization

## Funding

This research did not receive any specific grant from funding agencies in the public, commercial, or not-for-profit sectors.

## Declaration of Competing Interest

The authors declare that they have no known competing financial interests or personal relationships that could have appeared to influence the work reported in this paper.

## Data Availability

The data that support the findings of this study are publicly available at https://www.samhsa.gov/data/data-we-collect/teds-treatment-episode-data-set/datafiles

## Footnotes

1 Results including insurance status as a covariate demonstrated similar trends with a few exceptions (see Table A1 in the appendix). With this inclusion, rurality was not significant in the outpatient base model. Additionally, the interaction term between rurality and COVID was not significant in the RLT model.

2 Figure A1 of the appendix shows results for models including insurance status as a control variable. Conclusions drawn from the results are consistent with those shown in Fig. 3.
