# Supplementary material for: Rurality weakens the positive association between the COVID-19 pandemic and substance use treatment involving medications for opioid use disorder
Source: Drug Alcohol Depend Rep. 2026 Jan 5;18:100408. doi: 10.1016/j.dadr.2026.100408 (PMC12818227; doi:10.1016/j.dadr.2026.100408)
Supplement: Supplementary file 1 — Supplementary material [file mmc1.docx]

**Appendix**

| Table A1. Base and Moderation Logit Regression Models Analyzing Associations Between COVID, Rurality, and MOUD Use, Split by Treatment Modality, Controlling for Insurance Status | | | | | | |
| --- | --- | --- | --- | --- | --- | --- |
|  | Residential Short-Term | | Residential Long-Term | | Outpatient | |
|  | Base | Moderation | Base | Moderation | Base | Moderation |
| *Rural* | 0.180^***^ | 0.443^***^ | 0.535^***^ | 0.446^**^ | 0.973 | 1.009 |
|  | (0.150,0.217) | (0.348,0.563) | (0.406,0.705) | (0.270,0.738) | (0.945,1.002) | (0.973,1.047) |
| *COVID* | 3.222^***^ | 3.871^***^ | 3.039^***^ | 2.959^***^ | 1.159^***^ | 1.201^***^ |
|  | (2.871,3.616) | (3.419,4.382) | (2.547,3.626) | (2.457,3.564) | (1.126,1.193) | (1.159,1.244) |
| *Rural x Covid* |  | 0.204^***^  (0.142,0.293) |  | 1.301  (0.721,2.347) |  | 0.903^***^  (0.850,0.958) |
| *N* | 17,695 | 17,695 | 6,250 | 6,250 | 113,544 | 113,544 |
| Note: Odds Ratios Displayed; 95% confidence intervals in parentheses; Models adjusted for race and ethnicity, age, gender, education, employment, living arrangement, referral source, recent arrests, region, and insurance status  ^*^*p* < 0.05, ^**^ *p* < 0.01, ^***^ *p* < 0.001 | | | | | | |

Figure A1. Predicted Margins of MOUD Use by Year and Rurality, Split by Treatment Modality, Controlling for Insurance Status


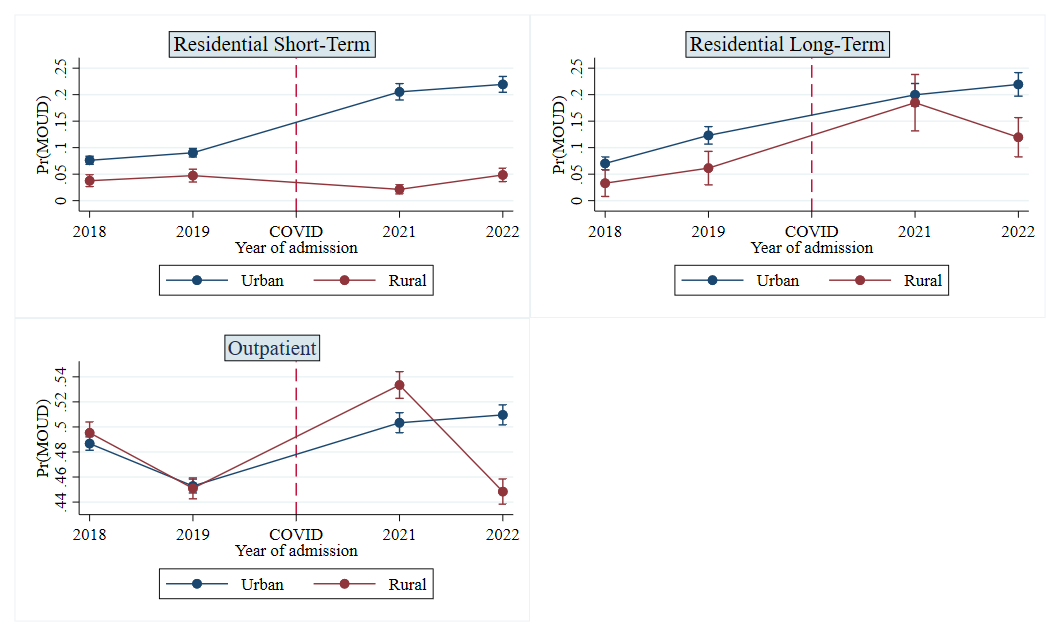


| Table A2. Moderation Logit Regression Models Analyzing Associations Between Year, Rurality, and MOUD Use, Split by Treatment Modality | | | |
| --- | --- | --- | --- |
|  | Residential Short-Term | Residential Long-Term | Outpatient |
| *Rural* | 0.415^***^ | 1.286^*^ | 0.826^***^ |
|  | (0.313, 0.550) | (1.010, 1.636) | (0.798, 0.856) |
| *Year (Base: 2018)* |  |  |  |
| *2019* | 1.366^***^ | 1.346^***^ | 0.948^***^ |
|  | (1.237, 1.508) | (1.161, 1.561) | (0.925, 0.972) |
| *2021* | 2.365^***^ | 2.772^***^ | 1.190^***^ |
|  | (2.130, 2.627) | (2.382, 3.225) | (1.154, 1.226) |
| *2022* | 2.740^***^ | 3.069^***^ | 1.232^***^ |
|  | (2.471, 3.039) | (2.636, 3.573) | (1.193, 1.273) |
| *Rural x 2019* | 0.934 | 0.802 | 1.125^***^ |
|  | (0.641, 1.362) | (0.566, 1.135) | (1.067, 1.185) |
| *Rural x 2021* | 0.318^***^ | 0.807 | 1.163^***^ |
|  | (0.204, 0.494) | (0.566, 1.150) | (1.094, 1.236) |
| *Rural x 2022* | 0.537^**^ | 0.363^***^ | 0.794^***^ |
|  | (0.364, 0.792) | (0.257, 0.513) | (0.747, 0.845) |
| *N* | 29,981 | 26,375 | 279,730 |
| Note: Odds Ratios Displayed; 95% confidence intervals in parentheses; Models adjusted for race and ethnicity, age, gender, education, employment, living arrangement, referral source, recent arrests, and region  **p* < 0.05, ^**^ *p* < 0.01, ^***^ *p* < 0.001 | | | |
